# Cancer Influences the Elemental Composition of the Myocardium More Strongly than Conjugated Linoleic Acids-Chemometric Approach to Cardio-Oncological Studies

**DOI:** 10.3390/molecules26237127

**Published:** 2021-11-25

**Authors:** Agnieszka Białek, Małgorzata Białek, Tomasz Lepionka, Anna Ruszczyńska, Ewa Bulska, Marian Czauderna

**Affiliations:** 1 Department of Biotechnology and Nutrigenomics, Institute of Genetics and Animal Biotechnology of the Polish Academy of Sciences, Postępu 36A Jastrzębiec, 05-552 Magdalenka, Poland; a.bialek@igbzpan.pl; 2 Department of Animal Nutrition, The Kielanowski Institute of Animal Physiology and Nutrition, Polish Academy of Sciences, Instytucka 3, 05-110 Jabłonna, Poland; m.bialek@ifzz.pl (M.B.); mr.czauderna@gmail.com (M.C.); 3 Department of Bioaerosols, The Biological Threats Identification and Countermeasure Center of the General Karol Kaczkowski Military Institute of Hygiene and Epidemiology, Lubelska 4 St, 24-100 Puławy, Poland; 4 Biological and Chemical Research Centre, Faculty of Chemistry, University of Warsaw, Żwirki i Wigury 101, 02-089 Warsaw, Poland; aruszcz@chem.uw.edu.pl (A.R.); ebulska@chem.uw.edu.pl (E.B.)

**Keywords:** chemometry, conjugated linoleic acid, breast cancer, heart, cardio-oncology

## Abstract

The aim of the study was to verify in a cardio-oncological model experiment if conjugated linoleic acids (CLA) fed to rats with mammary tumors affect the content of selected macro- and microelements in their myocardium. The diet of Sprague–Dawley females was supplemented either with CLA isomers or with safflower oil. In hearts of rats suffering from breast cancer, selected elements were analyzed with a quadrupole mass spectrometer with inductively coupled plasma ionization (ICP-MS). In order to better understand the data trends, cluster analysis, principal component analysis and linear discriminant analysis were applied. Mammary tumors influenced macro- and microelements content in the myocardium to a greater extent than applied diet supplementation. Significant influences of diet (*p* = 0.0192), mammary tumors (*p* = 0.0200) and interactions of both factors (*p* = 0.0151) were documented in terms of Fe content. CLA significantly decreased the contents of Cu and Mn (*p* = 0.0158 and *p* = 0.0265, respectively). The level of Ni was significantly higher (*p* = 0.0073), which was more pronounced in groups supplemented with CLA. The obtained results confirmed antioxidant properties of CLA and the relationship with Se deposition. Chemometric techniques distinctly showed that the coexisting pathological process induced differences to the greater extent than diet supplementation in the elemental content in the myocardium, which may impinge on cardiac tissue’s susceptibility to injuries.

## 1. Introduction

Noncommunicable diseases (NCDs), also known as chronic diseases, include cardiovascular diseases (CVD), cancers, chronic respiratory diseases and diabetes. They are the result of combination of genetic, physiological, environmental and behavioral factors [1]. CVD are the most common NCDs globally, responsible for an estimated 17.8 million deaths in 2017 [2], whereas cancer is a leading cause of death worldwide, accounting for an estimated 9.6 million deaths in 2018 [3]. Numerous authorities emphasize that CVD and cancer share the same risk factors, including tobacco smoking, physical inactivity, nutritional mistakes, alcohol consumption, obesity, hypertension and hyperlipidemia [4]. This evidence coupled with the fact that cardiotoxicity has become the main cause of morbidity and mortality of cancer survivors gave rise to the new “cardio-oncology” or “onco-cardiology” concept [5]. It includes early recognition, mitigation and prevention of the effects of cancer and its treatment on the cardiovascular system as well as the care of cancer patients with cardiovascular disease, overt or occult and already established or acquired during treatment.

Elements, together with water and other nutrients (proteins, fats, carbohydrates and vitamins) are considered crucial for human and animal life. Essential mineral elements include macroelements (Na, Mg, K and Ca), which constitute about 1.89%, while the rest is made up of 11 typical trace elements, which play key roles in metabolism from the lowest level of intracellular life to the functional activity of the largest organs and exert immense influence on all body functions organs. The composition of macro- and trace elements in body fluids and tissues is influenced by sex and age, dietary intake, uptake in the gastrointestinal tract, storage, excretion, physical condition and the presence or absence of disease state, as well as immune system disorders [6,7]. 

Both CVDs and cancer are the cause of disturbances in macro- and trace elements’ homeostasis in numerous tissues and organs. For example, in physiological conditions, myocardial mitochondria control the intracellular concentration of Ca ions, and pathologic changes in Ca homeostasis can initiate deleterious events leading to ischemic myocardial injury and cell death. The deposition of Ca salts into myocardial cells (dystrophic calcification) serves as a marker for heart muscle cell damage [8]. The morbid growth of mineral deposits in human heart valves (calcification) can lead to loss of their functionality and, thus, is the first reason for valve replacement and the third leading cause of CVD [9]. It was also established that trace elements, in particular Fe, Cu, Zn, Mn and Se, can affect the course of arterial hypertension and myocardial infarction [10]. Several elements (Se, Cr, Cu, Mg, Zn, Li and V) are suspected of being involved in the genesis of arteriosclerotic heart disease [11]. Cu and Fe are involved in many aspects of energy metabolism and are important in the synthesis of hemoglobin, myoglobin and cytochromes [12]. Se deficiency disturbs the optimal functioning of several cellular mechanisms and may promote the development of atherosclerosis [13]. Low Se concentration is also a risk factor for arterial hypertension and myocardial infarction, and a factor aggravating symptoms of myocardial ischemia [10]. Some trace elements modulate functions of cellular pro- and antioxidant systems [10], e.g., Cu, Zn and Mn protect against the effects of increased free reactive oxygen species via Cu-Zn superoxide dismutase and Mn superoxide dismutase [12]. This is of great importance, as the heart is prone to oxidative damage as it has lower activity of antioxidant enzymes (e.g., superoxide dismutase) than other tissues (e.g., liver) [14]. It was established that patients with heart failure have increased oxidative stress, which also causes a deficiency of Fe, Se and Zn [15]. Intensive production of reactive oxygen metabolites and activation of lipid peroxidation are the main mechanisms underlying myocardial damages [10] and increased lipid peroxidation in the myocardium is related to significant decrease in the heart content of Se, Mn and Cr [16]. Additionally, certain heavy metals (e.g., Cd, Co, Pb and Hg) can exert cardiotoxic effect due to, at least in part, increased vulnerability of the heart to free oxygen radicals [17]. 

Abnormalities in trace elements could severely impair an organism’s resistance against carcinogenic stress and induce carcinogenicity [18]. Trace elements may directly or indirectly affect the carcinogenic process, including tumor growth, replication, invasion and metastasis [19]. Some meta-analyses revealed that trace elements disturbances (Fe or Se deficiency) play an important role in carcinogenesis of breast tissue [20]. Taking into account that chemotherapy with adriamycin and cytoxan can significantly increase oxidative stress and decrease total antioxidant capacity in women with breast cancer [21], it may also influence elements content, especially those of antioxidant properties. It was noticed that elevated levels of Ca, Cr, Cu, Fe and Sr in breast cancer patients decreased and returned to the normal range after the chemotherapy. The opposite situation was observed for Ti, Zn and Se, as their levels restored to near normal range after chemotherapy [22]. On the one hand, metals and metal compounds are an important risk factor for the development of breast cancer, while on the other hand, they might have also beneficial effects, inducing apoptosis and cytotoxicity, in breast cancer cells [23]. Hence, knowledge about the imbalance of trace elements in biological samples of cancer patients might be helpful to elicit a unique possibility of identifying novel anti-tumor drugs based on the elements of significance [22].

Conjugated linoleic acid (CLA) isomers are a group of conjugated fatty acids (CFA) of multiple biological activities. They are positional and geometric isomers of linoleic acid (*c*9*c*12 C18:2) naturally present in different dietary sources of animal origin, especially meat and dairy products [24]. CLA isomers were established as potent anticancerogenic agents in a breast cancer model. Searching for the mechanisms of their action, they were found as forceful indicators of numerous metabolic changes in both physiological and pathological conditions, which may affect breast cancer risk [25,26,27,28,29,30,31]. These changes concerned the whole body, including the cardiac tissue. It was previously demonstrated that both diet supplementation with CLA isomers and coexisting cancerous process can influence the fatty acids profile and lipid peroxidation in cardiac tissue of female rats suffering from breast cancer [32]. Cancerous process intensified the lipid peroxidation (as confirmed by the elevated levels of 7-ketocholesterol in the hearts), while CLA isomers applied with the diet significantly inhibited polyunsaturated fatty acids (PUFA) oxidation (as evidenced by the lower content of malondialdehyde (MDA)). Taking into account that both dietary factors and cancerous process may influence macro- and trace elements content in the body (also in cardiac tissue), in the present study, a cardio-oncological approach is proposed for searching for other proposed mechanisms of CLA action. We hypothesized that dietary CLA isomers administrated to animals with mammary tumors may affect the elements’ composition in their hearts. The main aim of present study was to verify our hypothesis by evaluation of the influence of a commercial dietary supplement containing CLA isomers on the content of selected macro- and microelements in the hearts of female rats with chemically induced mammary tumors. Moreover, some correlations among investigated elements and fatty acids content, expressed by the indices related to CVD risk and lipid peroxidation markers, were investigated by using a chemometric approach.

## 2. Results

A detailed daily dietary intake of macro- and microelements is presented in Table 1. Their consumption as well as the consumption of other compounds was estimated based on the composition of ingredients of rats’ diets, assuming that 10 g of laboratory fodder and 0.15 ml of dietary supplement was ingested daily by a single rat. In Labofeed H, the most abundant macroelements were K, Ca, Mg and Na, which make them predominant in diets of experimental animals. In dietary supplements levels of elements were lower as compared to laboratory fodder: in SAF oil B, Al and Ca were detected in the highest amounts, whereas in Bio-C.L.A., only Ca, Ba, Cr, Pb and Sr were detected, as other elements were below the limit of detection (LOD). Diet modification with SAF oil or CLA supplement did not influence the mean dietary intake of investigated elements in all experimental groups (Table 1).

There were no spontaneous tumors during the experiment in groups not subjected to DMBA treatment. A single dose of intragastrically administrated DMBA was efficient in the induction of mammary tumors, identified as adenocarcinomas and papillary adenocarcinomas of the mammary gland. Detailed results concerning cancer incidence were presented previously [32].

The content of macroelements in cardiac tissue were affected by the presence of mammary tumors to a greater extent than by the animals’ diet (Table 2). This influence was observed for K, Mg and Na levels. As the same minerals are concerned, significant interactions of both experimental factors were observed simultaneously with a significant impact of tumors occurrence. 

Microelements contents in the rats’ hearts were only slightly influenced by the applied experimental factors (Table 2). Significant influences of diet (*p* = 0.0192), mammary tumors presence (*p* = 0.0200) and interactions of both examined factors (*p* = 0.0151) were documented in terms of Fe content in the hearts of rats. Supplementation of diet with CLA isomers also caused a significant decrease in the contents of Cu and Mn (*p* = 0.0158 and *p* = 0.0265, respectively) in cardiac tissue. The level of Ni in cardiac tissue of rats with mammary tumors was significantly higher (*p* = 0.0073), which was more pronounced in groups supplemented with Bio-C.L.A., where this increase was over fivefold. Among microelements, contents of Al and B and Tl were lower than the LOD (Table 2). The interaction of both examined factors also significantly influenced the quantity of the toxic heavy metal (Pb) (*p* = 0.0186), which was decreased synergistically by diet modification and mammary tumors presence. 

The Cu/Zn ratio was similar in all experimental groups and influenced neither investigated factors nor their interaction. The Mg/Ca ratio was the highest in SAF, whereas its lowest level was established in SAFplus. Significant differences were observed between these two groups. Both MT and D × MT influenced Mg/Ca values (*p* = 0.033 and *p* = 0.0186, respectively) (Table 2).

Despite similar daily intake of examined macro- and microelements of all groups, the chemometric approach applied in present study appeared as an objective strategy for evaluation and data interpretation of dissimilarities in mineral content in myocardium samples of experimental dietary groups, especially taking into account the coexisting pathological process that manifested as mammary tumor appearances. Different multivariate analyses were verified to represent a powerful method for distinguishing experimental groups on the basis of all of the existing dependencies. 

**Figure 1:**
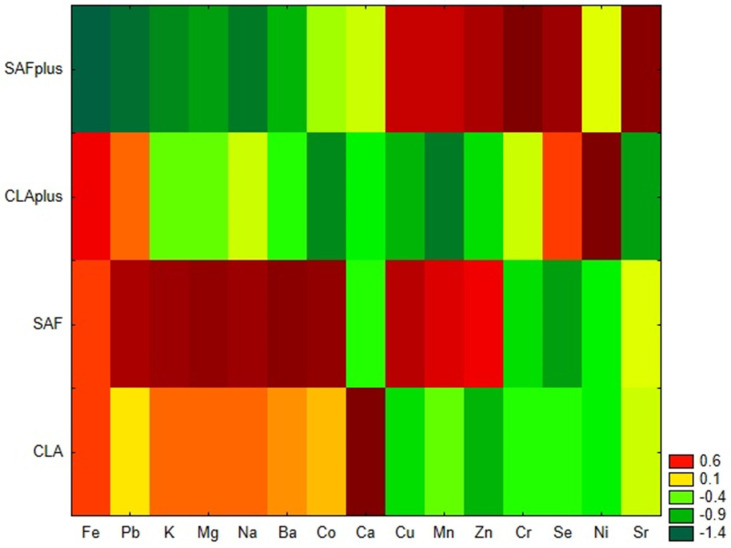
Heat maps of macro- and microelements content in the myocardium of experimental groups. SAF group—animals receiving safflower oil; SAFplus group—animals receiving safflower oil treated with DMBA; CLA group—group of animals receiving Bio-C.L.A.; CLAplus group—group of animals receiving Bio-C.L.A. treated with DMBA.

Similarity analysis, performed by the grouping of features and objects confirmed that both applied diet supplementation and the coexisting pathological process influence the mineral status of myocardium (Figure 1). SAFplus animals were characterized by a low content of Fe, Pb, K, Mg, Na and Ba and a high content of Cu, Mn, Zn, Cr Se and Sr. Unlike them, in cardiac tissue of rats receiving safflower oil, which were not subjected to DMBA treatment, high levels of Fe, Pb, K, Mg, Na and Ba, as well as Co, were observed. In myocardium of CLA-supplemented rats suffering from breast tumors, low levels of most of the examined minerals were detected. Only Fe, Pb, Se and Ni were present in high amounts. The CLA group without DMBA administration was characterized by a high content of Ca and low levels of Se and Ni, in comparison to the CLAplus group. 

**Figure 2:**
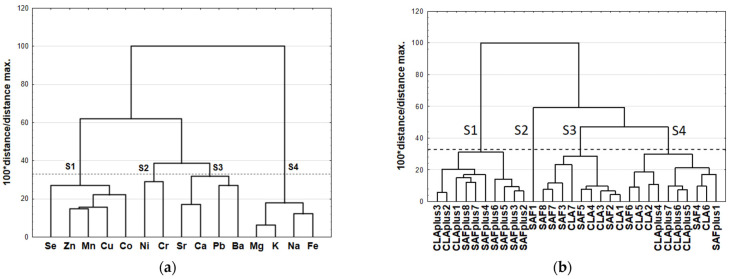
(**a**) Dendrogram of similarities of minerals and (**b**) their content in the myocardium of experimental groups. SAF group—animals receiving safflower oil; SAFplus group—animals receiving safflower oil treated with DMBA; CLA group—group of animals receiving Bio-C.L.A.; CLAplus group—group of animals receiving Bio-C.L.A. treated with DMBA.

The results of CA are presented as dendrograms in Figure 2a,b. The application Sneath’s criterion (33%) to the dendrogram analysis allowed us to distinguish four clusters (S1–S4) that group the examined minerals (Figure 2a). The first cluster (S1) included Co, Cu, Mn, Se and Zn, whereas Cr, Ni and Se were incorporated into the second cluster (S2). Ba, Ca, Pb and Sr created the third cluster and Fe, K, Mg and Na were included in the fourth cluster (S4). A dendrogram of the similarities in mineral content in myocardium samples revealed four clusters (S1–S4) due to the application of a more rigorous Sneath’s criterion (33%) (Figure 2b), but the samples’ allocation to distinguished clusters only coincided to a small extent with the experimental dietary groups. 

PCA analysis identified four factors carrying 80.9% of the total variability. The detailed matrix of factor analysis structure with the factor load values is presented in Table 3. The first principal component explained 30.5% of the total variance, whereas other PCs explained 28.1%, 14.3% and 8.0%, respectively. The highest contribution to the first principal component PC1 exhibited Fe, K, Mg and Na, whereas the highest contribution to PC2 exhibited Co, Cu, Mn and Zn. Ba, Ca, Pb and Sr gave their variances to PC3, and Ni and Se to PC4. The results of PCA are convergent with the results of CA. Statistical tests revealed the existence of significant differences between the values of three of the four principal components among the experimental groups (Table 4). 

**Figure 3:**
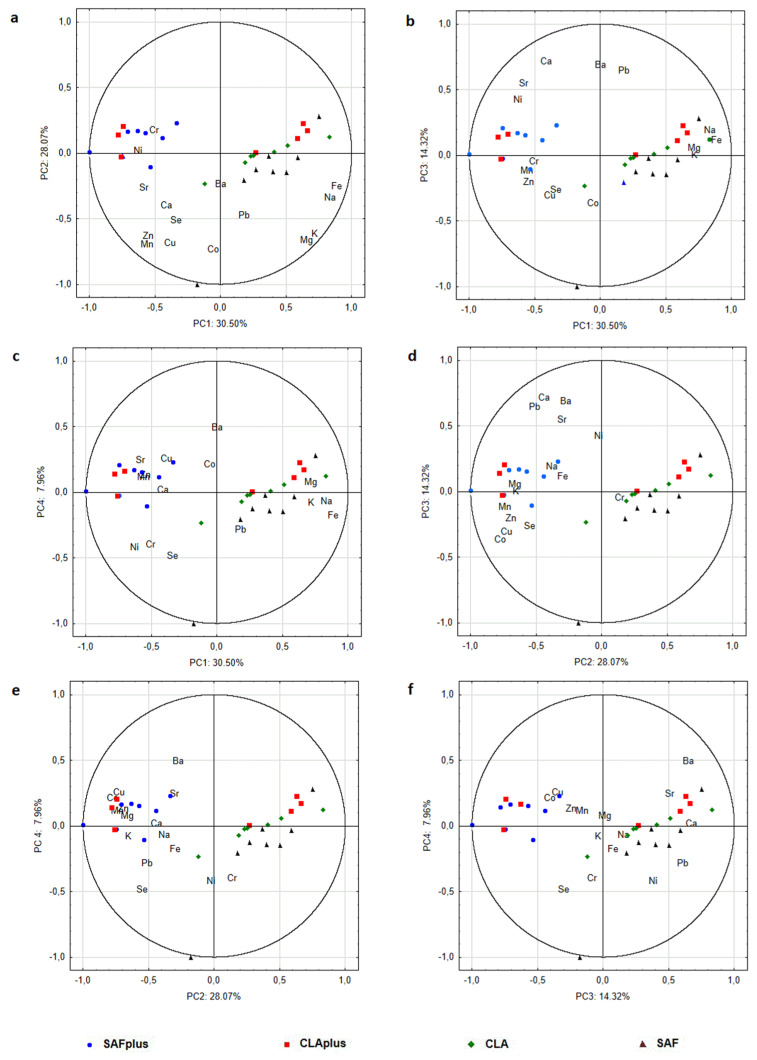
Biplots of minerals content in the myocardium of rats of experimental groups: (**a**) PC1 × PC2, (**b**) PC1 × PC3, (**c**) PC1 × PC4, (**d**) PC2 × PC3, (**e**) PC2 × PC4 and (**f**) PC3 × PC4. SAF group—animals receiving safflower oil; SAFplus group—animals receiving safflower oil treated with DMBA; CLA group—group of animals receiving Bio-C.L.A.; CLAplus group—group of animals receiving Bio-C.L.A. treated with DMBA.

Biplot PC1 × PC2 (Figure 3a) confirms that Na, Fe, Mg and K were positively correlated with the PC1 axis. Moreover, a strong positive correlation was revealed between Na and Fe as well as Mg and K. Zn, Mn, Cu and Co were strongly negatively correlated with the PC2 axis. However, a strong positive correlation was observed between Zn and Mn, and a little bit weaker positive correlation was shown for these two variables and Cu content. Individuals from SAF group were located in the positive values of PC1, which indicated high levels of Na, Fe, Mg and K, whereas SAFplus individuals were located in negative values of PC1 and were characterized by the lowest levels of these elements. PC1 is mainly responsible for the discrimination of SAF from SAFplus, whereas PC2 and PC4 are responsible for the separation of CLAplus from SAF. Complete distinction of all four dietary groups was not achieved by using PCA.

In the next step, LDA was used to obtain appropriate classification rules for the examined myocardium samples obtained from different experimental groups. Relevant discriminant functions were calculated in a stepwise progressive method. The percentage share of 15 minerals, which were detected in all examined myocardium samples, were included in the model. In the final model, eight variables were included, and four of them (Mg, Ni, Mn, Se) were significant in the model. All of them made a comparable contribution to overall discrimination. Applied canonical analysis allowed us to distinguish two statistically significant discriminant functions (DF). DF1 is the most significant function, as it explains 67.5% of the discriminatory power, whereas DF2 explains 27.2% of the discriminatory power (Table 5).

**Figure 4:**
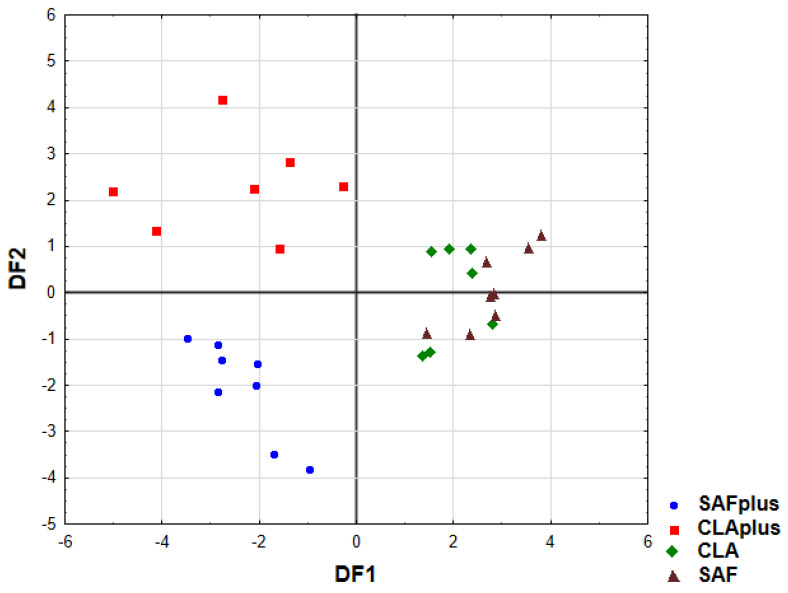
Scatter plot of canonical values for functions DF1 and DF2. SAF group—animals receiving safflower oil; SAFplus group—animals receiving safflower oil treated with DMBA; CLA group—group of animals receiving Bio-C.L.A.; CLAplus group—group of animals receiving Bio-C.L.A. treated with DMBA.

The analysis of canonical mean variables indicated that DF1 had the greatest impact on the distinction of CLAplus and SAFplus samples from other samples, whereas DF2 seemed to distinguish CLAplus from SAFplus samples (Table 5). Graph analysis confirms the suggestion provided by the values of average canonic variables (Figure 4).

A cross-validation of established LDA model was performed. Based on data concerning the content of the determined macro- and microelements, individuals were classified to experimental groups with the use of the discriminant functions distinguished in the first stage of LDA analysis. The calculated classification matrix indicated that the average classification efficiency based on the calculated functions was 93.3% (Table 6). For individual groups, these coefficients were as follows: 100% for SAFplus and CLAplus, respectively, 87.5% for SAF, and 85.7% for the CLA group.

**Figure 5:**
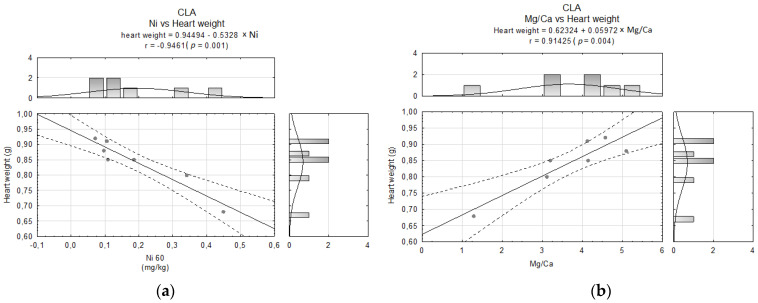
Correlations between (**a**) Ni content and heart weight and (**b**) Mg/Ca ratio and heart weight in the CLA group.

To give the most comprehensive view as possible, some dependences among the elements content and indices calculated based on fatty acids profile as well as heart weight were established. In the SAF group, the examined variables did not demonstrate any dependencies. Similarly, in the CLA group, only negative correlations between heart weight and Ni (Figure 5a) and heart weight and the Mg/Ca ratio (Figure 5b) were observed. 

**Figure 6:**
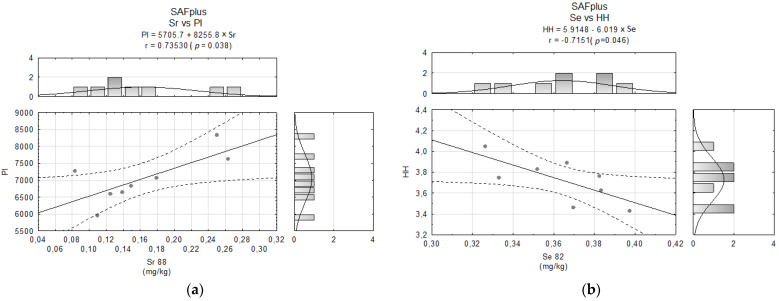
Correlations between (**a**) Sr content and peroxidability index and (**b**) Se content and HH index in the SAFplus group.

In the two examined groups afflicted by breast cancer, some interesting correlations were established. In the SAFplus group, a significant positive correlation was observed between Sr and the peroxidability index (PI) in the hearts of animals (Figure 6a). Hence, in this group, the hypo/hypercholesterolemic index (HH) was also negatively correlated with Se levels in cardiac tissue (Figure 6b). 

**Figure 7:**
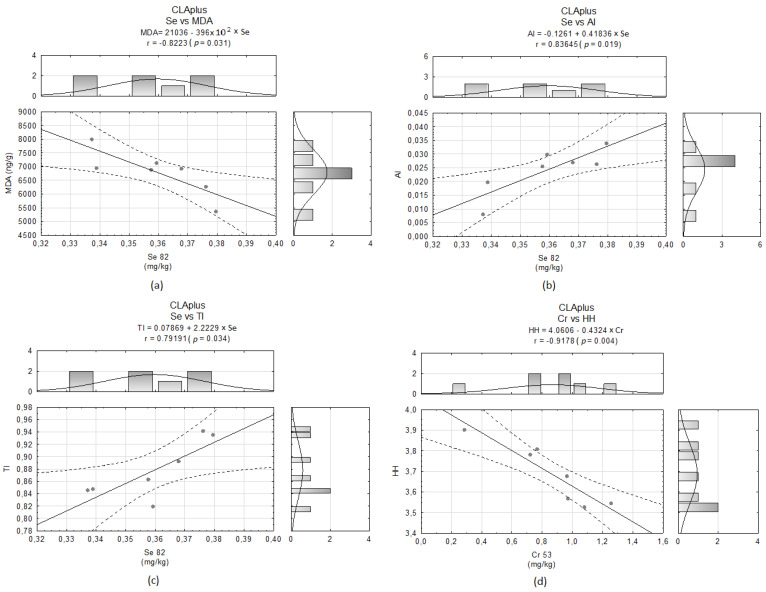
Correlations between (**a**) Se and malondialdehyde (MDA) content, (**b**) Se and AI, (**c**) Se and TI and (**d**) Cr and HH index in the CLAplus group.

Se levels in the hearts of animals from the CLAplus group demonstrated a significant negative dependence with MDA content (Figure 7a) and positive correlations with the indices of atherogenicity (AI) and thrombogenicity (TI) (Figure 7b,c, respectively) established on the fatty acids profile of the hearts of rats [32]. Cr amounts of the hearts in the CLAplus group were negatively correlated with the HH index (Figure 7d).

## 3. Discussion

Although cancer was previously considered to be one of the deadliest diseases, all applied medicinal procedures (e.g. screening, early detection or modern, targeted therapies) have made it a new “chronic” disease with an increasing number of survivors [33]. On the other hand, many chemotherapeutic agents, radiation therapies and novel, molecular therapies can negatively influence the cardiovascular system, causing cardiovascular mortality to be the principal cause of death 10 years after the diagnosis of breast cancer [34]. All these data coupled with the same risk factors shared by cancer and CVD contribute to the dynamic development of the cardio-oncology approach and to the investigation of different nutritional factors in cardio-oncological investigations. To the authors’ best knowledge, this is the first attempt to assess the influence of dietary CLA supplementation on macro- and microelements content in the myocardium in a cardio-oncological animal model experiment. 

The best established CLA influence on minerals is an increased intestinal Ca absorption, improved bone mass and reduced age-associated bone loss in mice [35]. Ca is also connected with CVD and cancer. In breast cancer tissue, microcalcifications are key diagnostically significant radiological features for localization of a malignancy [19], whereas in atherosclerosis, Ca depositions lead to the building up of plaque and consequently to narrowing or even blockage of the arteries [36,37]. In the present experiment, no differences in Ca levels in the myocardium of different experimental groups were detected, which indicates that CLA isomers’ influence may be limited only to Ca deposition in bones or in the liver [36,38]. 

Beside Ca, amounts of other macroelements were significantly reduced in the cardiac tissue of rats suffering from mammary tumors. Many studies have proven that those macroelements contribute to heart and muscle contractions, oxidative phosphorylation and the synthesis and activation of enzymatic systems [12]. Mg provides elasticity to prevent injury and acts with Ca to assist in muscle contraction and blood clotting and is thought to regulate blood pressure [39]. Deficiency of Mg contributes to tetany, Ca deficiency and the development of cardiovascular disease, diabetes mellitus and hypertension [39]. Significant decrease in serum concentration of Mg was observed in patients with breast cancer, especially those receiving chemotherapy [39]. The key role of decreased Mg concentration in pathogenesis of numerous disorders is linked with its capability to modulate intracellular processes via effects on signal transduction and antagonism of the effects of calcium (Ca^2+^) as a second messenger [40]. Decrease of intracellular Mg concentration results in impaired ATP utilization and causes intracellular imbalance of Ca and Na [40]. Not only absolute concentrations of Ca and Mg, but also their ratio is important for physiological regulation of different biochemical pathways [40]. A decreased Mg/Ca ratio was detected in rats suffering from mammary tumors, especially those receiving SAF oil. Mg content in the myocardium also appeared to be one of significant factors responsible for distinguishing the experimental groups in the LDA model. It confirms its meaning for cardiac tissue conditions, especially in cancer-suffering individuals.

Exposure to trace elements and their tissue concentration is considered to be a modifiable risk factor of many diseases [6]. At the normal range, neither in deficiency nor in excess, trace elements help maintain body homeostasis. Otherwise, they may contribute to breast cancer development by influencing oxidative DNA damage and endocrine and immune system problems [18]. Especially Fe acts as a catalyst for the production of reactive oxygen species. In patients with breast cancer, estrogen of blood circulation facilitates releasing Fe for ferritin storage, and released Fe stimulates oxidative stress in the breast tissue [21]. However, Fe levels in the serum of breast cancer patients were significantly lower than in healthy individuals [18], while increased Fe levels were determined in the serum of patients with acute coronary syndromes, which may have resulted from the release of this elements from ischemic tissue. In the present study, significantly decreased levels of Fe were observed in the myocardium of the SAFplus group, and both diet and the coexisting cancerous process influenced the Fe level in heart tissue.

Cu, similar to Fe, is considered an important factor in oxidative stress, which also participates in the pathogenesis of ischemic heart disease [41]. In the cell, Cu is mostly located in the cytosol, as a component of the superoxide dismutase, and in the mitochondria in proteins of the electron transfer chain, e.g., cytochromoxidase. Cardiac tissue, which is made up of cells with a high number of mitochondria, should be characterized by high Cu content [11]. In the case of Cu’s relation with breast cancer, some authors observed significantly reduced Cu levels in the serum of breast cancer patients [23,37,42], whereas others quantified its increased concentration in comparison with healthy subjects [6,7,18]. Moreover, levels of Cu in the tumor area were significantly higher than its levels found in the non-tumor tissue [19]. These diversified results indicate not only great dynamics of redox processes during carcinogenesis, but also Cu involvement as a cofactor of these processes in the cell. Cu produces ROS through activation of several structural peroxidases [21]. It also induces apoptosis by p53-dependent and -independent pathways [23]. Decreased levels of Cu detected in the myocardium of CLA-supplemented rats may suggest an influence of CLA dietary intake with Cu levels, and also may confirm our previous observation concerning antioxidant properties of CLA isomers [32]. Strong interactions between Cu and Fe as well as between Cu and Zn exist, e.g., changes in dietary Fe contents influence Zn and Cu status in the body [43]. Although some differences in both Fe and Cu content in the myocardium were detected in present study and numerous experiments emphasize Zn involvement in both cancer [7,19,21,23,37,42] and CVDs [7,11,14,41], no differences in Zn content were observed among experimental groups. It also reflected in lack of any differences in the Cu/Zn ratio in the present study, although according to some authors, the Cu/Zn ratio is higher in breast cancer patients [6,7].

It was observed that the serum level of Se reflects its content in heart tissue, whereas for other elements, such a strong relationship was not found [11]. It is of great importance that changes in the Se level in serum were observed for different parameters of heart work and markers of CVDs, e.g., Se showed a positive correlation with ejection fraction in patients with cardiomyopathy or coronary heart disease, or patients with coronary heart disease had lower serum and erythrocyte Se concentration than healthy controls [11]. Additionally, erythrocytes of patients with heart failure were deficient in Se [15]. Lower levels of Se correlated well with the degree of myocardial damage as indicated by high levels of the prognostic markers [41]. Similar to the case of Cu, the content of Se, which is part of the glutathione peroxidase system in the cytosol and mitochondria, is high in heart muscle cells [11]. In rats with hereditary stress-induced arterial hypertension, Se contents in the plasma and myocardium were higher than in normotensive animals, which was related to antioxidant properties of Se. Dietary Se is essential for the biosynthesis of Se enzymes (especially Se-cysteine-containing enzymes) and lipid antioxidants, and free Se-containing amino acids produce antioxidant effects, since they scavenge free radicals and are involved in non-radical degradation of lipid peroxides. In the rat myocardium, Se-containing peptides possessing antioxidant properties and catalyzing the indirect degradation of superoxide radicals were detected. All of these features make Se a potent cardioprotector [10]. Additionally, a strong inverse relationship has been found between serum Se concentration and risk of breast cancer, as Se exerts an anti-neoplastic effect as an important component of the antioxidant system (e.g., glutathione peroxidases containing Se-cysteine) [6]. Although no differences in Se content among experimental groups were observed in the present study, some interesting dependences among Se content and lipid peroxidation as well as indices of the fatty acids profile were observed. It is noteworthy to indicate that Se levels in cardiac tissue of the CLAplus group demonstrated a significant negative dependence with MDA content. This confirms antioxidant properties of Se and suggests antioxidant properties of CLA isomers [32] as well as an interaction between CLA and Se content in tissues, which we observed previously [44]. However positive correlations among Se and AI and TI reveal the strong need for further research concerning CLA and Se interactions as well as their health implication in both physiological and pathological conditions. It also confirms our strong belief that there is a great need of clear delimitation of drugs and dietary supplements and a great need of extensive investigations, as dietary supplements can exert opposite effects when given in physiological and pathological conditions.

Mn might share the similar anticarcinogenic properties as Fe and Se. Similar to Mg, Se and Mn contents in the myocardium also appeared to be significant factors responsible for distinguishing the experimental groups in the LDA model. It confirms the great importance of Se and Mn levels for cardiac tissue conditions, especially in cancer-suffering individuals, especially due to their engagement in counteracting oxidative stress. Mn is a functional cofactor of various enzymes crucial for the various cellular activities, e.g., manganese superoxide dismutase (Mn-SOD) contains Mn in its active site, which has the cancer-fighting properties. High intracellular Mn can compensate for the loss of SOD and provide protection against oxidative stress. Mn disturbance might increase the risk of breast cancer through disrupting the balance of the oxidant/antioxidant system. Its lower levels were detected in breast cancer patients [20]. However, according to Choi et al., serum Mn levels were significantly higher (*p* < 0.05) in breast cancer patients than in controls patients [45]. Mn levels were decreased in the myocardium of rats from both CLA-supplemented groups, which indicates some dependencies between Mn and CLA, which need further examinations.

Numerous research studies have also revealed the involvement of some toxic elements in the cancerous process. Cd and Ni are classified in human carcinogen group 1 by the World Health Organization. Cd inhibits DNA synthesis, mismatch repair and enzyme function [23,46]. Ni compounds with the inhibition of intercellular connection could exhibit tumor-promoting ability through some mechanisms, such as immortalization of epithelial cells, generation of DNA protein cross-links, blockage of nucleotide-cutting repair and an increase in gene expression through DNA methylation [46]. Its serum concentration was significantly increased in breast cancer patients receiving chemotherapy [39]. In the present study, increased Ni content was detected in the myocardium of rats with mammary tumors, which confirms the previous results. Its content in cardiac tissue was also one of the significant factors responsible for distinguishing the experimental groups in the LDA model. Co is involved in DNA breaks and the inhibition of DNA repair and can be considered as a carcinogen. In physiological concentration, it acts in a cardioprotective manner, while its high dosage may cause cardiac failure [39]. Co content in the myocardium did not differ among experimental groups in the present study. Zheltova et al. found that Mg deficiency provoked changes of Co concentration in the most of studied tissues (in the heart, aorta, liver, kidneys, spleen and ovaries) and a 2–3 -old decrease of the Ni level in the heart, thyroid gland, spleen and uterus compared to the physiological level. They speculated that some Mg-containing transporters may contribute to Co and Ni transport through biological membranes [40]. 

## 4. Materials and Methods

### 4.1. Dietary Ingredients

Laboratory fodder Labofeed H was purchased from “Morawski” Feed and Concentrates Production Plant (Kcynia, Poland). Commercially available Bio-C.L.A. dietary supplements in the form of gel capsules, containing an equimolar mixture of *c*9*t*11CLA and *t*10*c*12CLA, as well as safflower oil (SAF oil), used as substrate for Bio-C.L.A. production, were obtained free of charge by Pharma Nord (Warsaw, Poland). The composition of dietary ingredients in detail was published elsewhere [32].

### 4.2. Animal Experiment

This study was approved by the 2nd Local Ethical Committee on Animal Experiments at Medical University of Warsaw in terms of guiding principles on the care and use of laboratory animals (No. 34/2008). Virgin female Sprague–Dawley rats (*n* = 46, 30 days old) were obtained from the Division of Experimental Animals, Department of General and Experimental Pathology (Medical University of Warsaw, Warsaw, Poland). They were housed at 21 °C, in a 12 h light: 12 h dark cycle in a conventional animal room with constant access to the laboratory fodder and fresh drinking water ad libitum during the entire experiment. After 1 week of adaptation, the animals were randomly divided into 4 experimental groups. Detailed characteristics of the experimental groups are presented below.

SAF group (*n* = 8) and SAFplus group (*n* = 14)—animals were fed laboratory fodder and received 0.15 ml of SAF oil daily via gavage.

CLA group (*n* = 7) and CLAplus (*n* = 17)—animals were fed laboratory fodder and were given 0.15 ml of commercial dietary supplement daily (Bio-C.L.A.) via gavage. A detailed daily intake of dietary ingredients is given in Table 1.

Animals of each of the two “plus” groups intragastrically received in the 50th day of life a single dose (80 mg/kg body weight) of chemical carcinogen: 7,12-dimethylbenz[a]anthracene (DMBA, approx. 95%; Sigma-Aldrich, Saint Louis, Missouri, USA), which was previously dissolved in SAF oil (SAFplus group) or in the oily filling pressed out from the capsule of commercial supplement (CLAplus group). Rats from SAF and CLA groups were not treated with DMBA. The dietary intervention lasted from the 37^th^ day of life of animals for the subsequent 21 weeks. Due to the unknown morbidity, DMBA-treated groups (SAFplus and CLAplus) were more numerous in comparison to groups without DMBA treatment (SAF and CLA) to ensure a statistically adequate number of rats with tumors. 

Animals were monitored daily by the experienced veterinarian for specific signs of welfare and health disorders and weekly weighed and palpated for evaluation of tumors appearance. All the rats were decapitated in week 21 of the experiment and after exsanguination their hearts were excised, weighed (wet weight) and stored frozen in −80 °C for further analyses. The experiment design as well as data concerning the hearts’ mass was previously presented in detail in Figure 1 and in Table 2 [32] and they were not repeated in this manuscript to avoid self-plagiarism.

### 4.3. ICP-MS Elements Determination

The content of elements in hearts as well as in fodder and dietary supplements were determined according to Jagielska et al. [47]. Samples were dried for 24 h in 37 °C in the drying oven SLN 240 (Pol-Eko, Wodzislaw Slaski, Poland), then weighed (100 mg–300 mg) and subjected to mineral digestion in the 69% aqueous solution of nitric acid (analytical grade, Merck, Darmstadt, Germany) (1:2, *v*/*v*) in glass tubes of a microwave ultraWave closed system with single reaction chamber technology (Milestone, Milan, Italy) for 15 min up to 200 °C and 10 min in 200 °C. After cooling down to the ambient temperature, digests were diluted in deionized water. For isotope-specific detection of selected elements (^11^B, ^23^Na, ^24^Mg, ^27^Al, ^39^K, ^42^Ca, ^57^Fe, ^53^Cr, ^55^Mn, ^59^Co, ^60^Ni, ^63^Cu, ^66^Zn, ^82^Se, ^88^Sr, ^111^Cd, ^138^Ba, ^205^Tl, ^208^Pb), a quadrupole mass spectrometer with inductively coupled plasma ionization, ICP-MS (Nexion 300D, Perkin Elmer Sciex, Waltham, Massachusetts, USA), equipped in quartz cyclonic spray chamber, Meinhard nebulizer and platinum skimmer cones, was used. The working conditions of the spectrometer were as follows: radio frequency plasma power −1350 W and the constant nebulizer gas (Ar) flow −0.9 dm^3^/min. Transient signals of the selected isotopes were monitored (1 reading/5 sweeps/3 replicates) with a dwell time of 100 ms/isotope. Quantitation was achieved by 5-point external calibration (concentration range: 1 μg/dm^3^–100 μg/dm^3^). The limits of detection (LOD) and limit of quantitation (LOQ) were calculated for each element according to the IUPAC recommendation [48] together with the recoveries presented in the Table 7. Recoveries were calculated as a ratio of received to certified values and presented as percentages. Samples and standards (ICP multi-element standard Merck VIII (Merck, Darmstadt, Germany), SRM 1577c Bovine Liver (NIST, USA)) were diluted with deionized water obtained by the Milli-Q system (Merck, Millipore, Darmstadt, Germany).

### 4.4. Malondialdehyde Analysis

Samples of hearts were subjected to gentle alkaline saponification and derivatization with 2,4-dinitrophenylhydrazine (DNPH), followed by extraction with hexane and subjected to high-performance liquid chromatography analysis. The detailed methodology and results have been published previously [32].

### 4.5. Indices Attributed to Calculation of Fatty Acids Properties 

Fatty acids content in cardiac tissue was quantified with capillary gas chromatography coupled with mass spectrometry (GC-MS). On the basis of the fatty acids content, indices attributed to the selected properties of fatty acids (index of atherogenicity—AI, index of thrombogenicity— TI, hypo/hypercholesterolemic index—HH) were calculated. The detailed methodology and results have been published previously [32].

### 4.6. Statistical Analysis

The results, presented as means ± standard deviation (SD), were analyzed with Statistica 13 software [49]. For the elimination of outstanding results, Dixon’s Q test was used, while for the verification of normality of data distribution, the Shapiro–Wilk test was used. Only tumor-bearing rats from the SAFplus and CLAplus groups were considered in statistical analyses. Before statistical analyses, data with skew distribution were log-transformed. Multi-factor analysis of variance (ANOVA) was used to evaluate effects of diet (D), presence of mammary tumors (MT) and their interactions (D × MT). When interaction occurred (*p* ≤ 0.05), the significances of differences among groups were established using the post hoc HSD Tukey test for uneven numbers for variables with normal distribution or the multiple comparison test for variables with skew distribution. *p* ≤ 0.05 was considered significant.

In order to better understand the data trends, chemometric procedures were applied, where mineral levels in the myocardium were used as chemical descriptors to study a possible discrimination of the experimental groups. Because of missing data, the following elements—Al, B and Tl—were excluded from the analyses. Prior to all chemometric analyses, the original data were transformed into natural logarithms (log-transformed) and then auto-scaled (standardized) to avoid the dominant influence of macroelements on the prepared models. Only tumor-bearing rats from the SAFplus and CLAplus groups were considered in statistical analyses. Similarity analysis was performed by grouping of features and objects to prepare a heat map. Cluster analysis (CA) was performed to determine the similarity of the examined variables. Hence, CA was also performed to determine the similarity of the examined biological samples described by the set of variables. These analyses were carried out using the agglomeration method. Moreover, the Euclidean distance was used as the distance determination method and the Ward method was used as the agglomeration method. The application of more restrictive Sneath’s criterion (33%) was used for the dendrograms analysis and distinguishing clusters. Next, in order to provide a first evaluation of discriminating efficiency of mineral content in the myocardium, principal component analysis (PCA) was performed with standardized Varimax rotation. The factors were distinguished based on eigenvalues greater than 1. The calculated factor values were compared between the groups using the Kruskal–Wallis test and multiple comparison test with the assumed significance level *p* ≤ 0.05. Finally, in order to obtain appropriate classification rules for the myocardium samples based on the mineral levels, a linear discriminant analysis (LDA) was performed. In discriminant analysis, two essential steps are distinguished: In the first (learning) stage the classification rules are searched, creating of analytical model and the establishment of discriminant functions. In the second stage, which is called the classification (or validation) stage, based on rules defined in the learning stage, a set of objects, the belonging of which is unknown, is classified. In order to optimize LDA, relevant discriminant functions were calculated in a stepwise progressive method, with the adopted tolerance value 1 − R^2^ = 0.01. The classification (validation) stage can be performed in two variants: (1) post hoc, when an object is classified to the group using the discriminant functions, which were established including this object (internal validation); or (2) a priori, when an object is classified to the group using the discriminant functions, which were established based on prior analysis of other objects (external validation). In our manuscript, due to the fact that no new set of objects (animals of experimental groups) was available, we performed the internal validation of our established LDA model in the post hoc variant. Based on data concerning the content of determined macro- and microelements, individuals were classified to experimental groups with the use of discriminant functions distinguished in the first stage of LDA analysis. The results of this cross-validation procedure, which describe the effectiveness of case (individuals) classification to experimental groups based on the previously created classification functions, was shown as the percentage of correct classification.

Hence, Spearman’s coefficients were calculated for elements content and heart mass as well as for elements content and previously presented indices [32] attributed to selected properties of FAs, calculated on the basis of the FA profiles in cardiac tissue. *p*-value ≤ 0.05 was considered significant.

## 5. Conclusions

The obtained results emphasize that dependencies among different elements in the body are complex and many different factors, such as diet, dietary supplements and physiological or pathological state, may influence them. All applied chemometric techniques, especially linear discriminant analysis, distinctly showed that the coexisting pathological process, to a greater extent than the applied diet supplementation with CLA or SAF, influences macro and microelements content in cardiac tissue. Induced changes include pronounced differences in macro- and microelements content in the myocardium of rats suffering from mammary tumors, which in turn may impinge on cardiac tissue condition and its susceptibility to injuries. It is of great importance, especially for oncological patients, to monitor the minerals content, especially taking into account possible cardiologic complications. The obtained results also confirmed the antioxidant properties of CLA and their relationship with Se deposition.

## Figures and Tables

**Table 1:** Daily intake of dietary ingredients.

	Diet	
	SAF/SAFplus	CLA/CLAplus
**Elements:**		
K [mg]	<186	<186
Mg [mg]	<29.3	<29.3
Na [mg]	<24.2	<24.2
Ca [mg]	76.9	76.9
Fe [mg]	<2.27	<2.27
Zn [mg]	<0.34	<0.34
Cu [mg]	<0.10	0.10
Mn [mg]	0.43	<0.43
Se [μg]	1.65	<1.65
Co [μg]	2.25	2.25
Cr [μg]	3.00	2.99
Ni [μg]	10.2	10.2
Al [mg]	1.52	<1.51
Sr [mg]	0.11	0.11
Ba [μg]	53.7	53.6
Pb [μg]	1.78	1.78
**Fatty acids:**		
C6:0 [μg]	104	104
C8:0 [mg]	0.00	0.17
C10:0 [μg]	0.00	127
C12:0 [μg]	48.0	52.0
C14:0 [μg]	215	215
C15:0 [μg]	107	100
C16:0 [mg]	12.4	12.7
*c*7 C16:1 [μg]	142	123
*c*9 C16:1 [μg]	201	196
C17:0 [μg]	108	111
*c*6 C17:1 [μg]	62.0	62.0
*c*9 C17:1 [μg]	9.52	7.48
C18:0 [mg]	4.40	5.29
*t*11 C18:1 [μg]	0.00	7.37
*c*9 C18:1 [mg]	29.1	16.3
*c*11 C18:1 [mg]	0.69	0.77
*t*9*c*12 C18:2 [μg]	0.00	27.9
*c*9*c*12 C18:2 [mg]	51.6	47.1
*c*9*c*12*c*15 C18:3 [mg]	22.2	22.1
C20:0 [μg]	237	218
*c*9*t*11 C18:2 [mg]	0.00	13.7
*t*7*c*9 C18:2 [μg]	0.00	130
*t*10*c*12 C18:2 [mg]	0.00	13.5
*c*11*c*13 C18:2 [mg/g]	0.00	0.57
*c*9*c*11 C18:2 [μg]	0.00	96.5
*c*11 C20:1 [mg]	189	104
*c*8*c*11*c*14*c*17 C20:4 [μg]	92.7	0.00
C22:0 [μg]	54.0	104
C24:0 [μg]	27.9	8.96
*c*15 C24:1 [μg]	33.1	25.8
**Conjugated fatty acids:** [mg/g]		
ƩCFA:	0.07	26.5
ƩCD:	0.03	26.1
*tt* CD	0.02	0.71
*ct/tc* CD	0.01	24.6
*cc* CD	0.00	0.86
ƩCT:	0.04	0.41
*ttt* CT	0.03	0.38
*ttc* CT	0.00	0.03
*cct* CT	0.00	0.00
**Cholesterol** [μg]	1550	1550
**Tocopherols:** [μg]		
δ (delta) tocopherol	278	281
γ (gamma) tocopherol	46.9	47.2
α (alpha) tocopherol	150	173
α (alpha) tocopherol acetate	842	831

CFA—conjugated fatty acids, CD—conjugated dienes, CT—conjugated trienes, *cc*—*cis,cis* isomers, *ct*/*tc*—*cis,trans*/*trans,cis* isomers, *tt*—*trans,trans* isomers, *ttt*—*trans,trans,trans* isomers, *ttc*—*trans,trans,cis* isomers, *cct*—*cis,cis,trans* isomers.

**Table 2:** Content of macro- and microelements in hearts of female rats supplemented with safflower oil (SAF oil) or Bio-C.L.A.

Diet		SAF Oil		Bio-C.L.A.		*p* Values for Two-Way ANOVA		
	Group	SAF (*n* = 8)	SAFplus (*n* = 14)	CLA (*n* = 7)	CLAplus (*n* = 17)	Diet (D)	Mammary Tumors (MT)	Interaction (D × MT)
Variables								
**Macroelements:**								
**K**		6048 ± 1588 ^c^	3258 ± 378 ^a^	5061 ± 316 ^abc^	4148 ± 1186 ^ab^	0.8992	<0.0001	0.0197
**Mg**		471 ± 121 ^c^	233 ± 23 ^a^	380 ± 9 ^bc^	295 ± 79 ^ab^	0.5913	<0.0001	0.0097
**Na**		1923 ± 541 ^b^	1046 ± 228 ^a^	1502 ± 135 ^a^	1775 ± 207 ^ab^	0.6589	0.0001	0.0287
**Ca**		103 ± 23	106 ± 29	124 ± 75	102 ± 26	0.5861	0.5257	0.4195
**Microelements:**								
**Fe**		163 ± 42 ^b^	46.8 ± 0.6 ^a^	160 ± 21 ^b^	287 ± 97 ^b^	0.0192	0.0200	0.0151
**Zn**		16.4 ± 4.1	16.7 ± 1.4	14.9 ± 0.5	15.1 ± 2.9	0.1103	0.7854	0.9451
**Cu**		8.29 ± 2.37	8.19 ± 1.09	6.99 ± 0.63	6.85 ± 0.36	0.0158	0.8155	0.9582
**Mn**		0.72 ± 0.23	0.73 ± 0.11	0.63 ± 0.05	0.58 ± 0.08	0.0265	0.6594	0.5286
**Se**		0.35 ± 0.09	0.36 ± 0.03	0.35 ± 0.02	0.36 ± 0.02	0.9645	0.6280	0.8576
**Co**		0.14 ± 0.05	0.12 ± 0.02	0.13 ± 0.01	0.11 ± 0.02	0.2190	0.0618	0.8339
**Cr**		0.41 (0.17–0.98) ^#^	1.20 (0.56–2.56) ^#^	0.74 ± 0.71	0.86 ± 0.31	0.4176	0.1924	0.2989
**Ni**		0.21 ± 0.11	0.40 ± 0.23	0.19 ± 0.15	0.99 ± 0.91	0.1069	0.0073	0.0863
Al *		<111	<111	<111	<111	-	-	-
**Sr**		0.13 ± 0.03	0.16 ± 0.06	0.15 ± 0.07	0.13 ± 0.04	0.1925	0.7278	0.3091
**Ba**		0.72 ± 0.08	0.33 ± 0.20	1.04 ± 0.00	0.65 ± 0.00	0.6331	0.2908	0.5708
**Pb**		0.11 ± 0.04 ^b^	0.09 ± 0.05 ^a^	0.09 ± 0.04 ^ab^	0.08 ± 0.03 ^ab^	0.4046	0.0880	0.0186
B *		<234	<234	<234	<234	-	-	-
Tl *		<0.009	<0.009	<0.009	<0.009	-	-	-
**Ratios:**								
**Cu/Zn**		0.50 ± 0.03	0.49 ± 0.05	0.47 ± 0.04	0.46 ± 0.06	0.1033	0.5612	0.8618
**Mg/Ca**		4.65 ± 0.94 ^a^	2.33 ± 0.59 ^b^	3.65 ± 1.25 ^ab^	3.25 ± 1.66 ^ab^	0.9258	0.0033	0.0316

* content was below the limit of detection (LOD): 111 mg/kg for Al, 234 mg/kg for B and 0.009 mg/kg for Tl; data are shown as mean values ± standard deviation (SD). Variables with skew distribution were transformed into logarithms, retransformed after calculations and presented as mean and confidence interval and marked with ^#^. *p* value ≤ 0.05—significant differences among groups in two-way ANOVA. 0.00—amount was below the quantification limit (<LOQ). D—diet; MT—mammary tumors, D × MT—interaction; when interaction (D × MT) occurs, the significance of differences among groups was further analyzed by post hoc HSD Tukey test or multiple comparison tests. abc—values with different superscripts in rows significantly differ at *p* value ≤ 0.05. SAF group—animals receiving safflower oil; SAFplus group—animals receiving safflower oil treated with DMBA; CLA group—group of animals receiving Bio-C.L.A.; CLAplus group—group of animals receiving Bio-C.L.A. treated with DMBA.

**Table 3:** Loadings, eigenvalues and variances of the significant principal components.

	PC1	PC2	PC3	PC4
Fe	**0.8856**	−0.2961	0.0761	−0.2164
K	**0.7145**	−0.6576	−0.0385	−0.1210
Mg	**0.7140**	−0.6606	0.0144	0.0365
Na	**0.8338**	−0.3819	0.1516	−0.1083
Ba	−0.0009	−0.2755	**0.6476**	0.4499
Ca	−0.4113	−0.4392	**0.6750**	−0.0232
Co	−0.0576	**−0.7770**	−0.4085	0.1693
Cr	−0.5076	0.1352	−0.0874	−0.4381
Cu	−0.3889	**−0.7254**	−0.3506	0.2119
Mn	−0.5605	**−0.7384**	−0.1584	0.0714
Ni	−0.6278	−0.0261	0.3796	**−0.4596**
Pb	0.1769	−0.5156	**0.6041**	−0.3285
Se	−0.3400	−0.5530	−0.3038	**−0.5275**
Sr	−0.5850	−0.3041	**0.5078**	0.2025
Zn	−0.5447	**−0.6941**	−0.2456	0.0885
Eigenvalue	4.58	4.21	2.15	1.19
Variance (%)	30.5	28.1	14.3	7.96
Cumulative (%)	80.9			

The most significant loadings are boldfaced.

**Table 4:** The comparison of experimental groups regarding identified factors.

	SAF	SAFplus	CLA	CLAplus	*p* Value
PC1	0.63 ± 0.50 ^b^	−1.10 ± 0.36 ^a^	0.58 ± 0.53 ^b^	−0.04 ± 1.25 ^ab^	0.0092
PC2	−0.76 ± 1.56 ^a^	0.38 ± 0.50 ^ab^	−0.10 ± 0.49 ^ab^	0.52 ± 0.42 ^b^	0.0159
PC3	0.10 ± 1.02	−0.50 ± 0.92	0.06 ± 1.34	0.38 ± 0.56	0.2668
PC4	0.52 ± 1.18 ^a^	0.33 ± 0.75 ^ab^	0.02 ± 0.57 ^ab^	−0.98 ± 0.77 ^b^	0.0229

Data are presented as mean ± SD. ab—values with different superscripts in rows significantly differ at *p* value ≤ 0.05 multiple comparison test. SAF group—animals receiving safflower oil; SAFplus group—animals receiving safflower oil treated with DMBA; CLA group—group of animals receiving Bio-C.L.A.; CLAplus group—group of animals receiving Bio-C.L.A. treated with DMBA.

**Table 5:** Coefficients and average value of canonical variables included in the final model.

Coeffcients of Canonical Variables		
Variable (Discriminatory Power)	DF1 (67.5%)	DF2 (27.2%)
Mg	1.32041	0.95449
Cu	−1.18252	−0.30459
Ni	−1.30381	1.44142
Mn	1.31693	−1.66926
Se	−0.89319	0.36417
Pb	0.52141	0.37002
Co	0.65777	0.53331
Ca	0.31885	−0.22727
**Avarage value of canonical variables**		
SAF	2.77985	0.06798
SAFplus	−2.34742	−2.06203
CLA	1.98196	−0.02354
CLAplus	−2.47616	2.30245

**Table 6:** Classification results of the LDA presenting the percentage of predicted group membership for actual groups.

Actual Group	Correct Classification (%)	Predicted Group Membership			
		SAF	SAFplus	CLA	CLAplus
SAF	87.5	0	0	1	7
SAFplus	100.0	8	0	0	0
CLA	85.7	0	0	6	1
CLAplus	100.0	0	7	0	0
Σ	93.3	8	7	7	8

SAF group—animals receiving safflower oil; SAFplus group—animals receiving safflower oil treated with DMBA; CLA group—group of animals receiving Bio-C.L.A.; CLAplus group—group of animals receiving Bio-C.L.A. treated with DMBA.

**Table 7:** Values of limit of detection (LOD) (mg/kg), limit of quantification (LOQ) (mg/kg) and recovery (%) of detected elements.

	LOD	LOQ	Recovery
K	91.93	105.6	135
Mg	8.93	16.62	104
Na	644.4	829.2	130
Ca	21.47	26.06	90
Fe	70.51	82.71	80
Zn	0.202	0.325	85
Cu	0.063	0.090	90
Mn	0.023	0.028	101
Se	0.008	0.012	85
Co	0.001	0.001	103
Cr	0.035	0.043	400
Ni	0.024	0.033	114
Al	110.8	134.0	-
Sr	0.077	0.079	115
Ba	0.573	0.594	-
Cd	0.000	0.000	85
Pb	0.046	0.051	115
B	234.4	270.6	-
Tl	0.009	0.013	-

## Data Availability

Data available on request. The data presented in this study are available on request from the corresponding author. The data are not publicly available due to privacy restrictions of the collaborators.

## Sample Availability

Samples of the PSO and BME or samples of biological material are not available from the authors.
